# Class Energy Image Analysis for Video Sensor-Based Gait Recognition: A Review

**DOI:** 10.3390/s150100932

**Published:** 2015-01-07

**Authors:** Zhuowen Lv, Xianglei Xing, Kejun Wang, Donghai Guan

**Affiliations:** 1 College of Automation, Harbin Engineering University, Harbin 150001, China; 2 Department of Computer Engineering, Kyung Hee University, Seoul 130-701, Korea; E-Mail: donghai@oslab.khu.ac.kr

**Keywords:** gait recognition, gait representation, Class Energy Image

## Abstract

Gait is a unique perceptible biometric feature at larger distances, and the gait representation approach plays a key role in a video sensor-based gait recognition system. Class Energy Image is one of the most important gait representation methods based on appearance, which has received lots of attentions. In this paper, we reviewed the expressions and meanings of various Class Energy Image approaches, and analyzed the information in the Class Energy Images. Furthermore, the effectiveness and robustness of these approaches were compared on the benchmark gait databases. We outlined the research challenges and provided promising future directions for the field. To the best of our knowledge, this is the first review that focuses on Class Energy Image. It can provide a useful reference in the literature of video sensor-based gait representation approach.

## 1. Introduction

**Figure 1.**
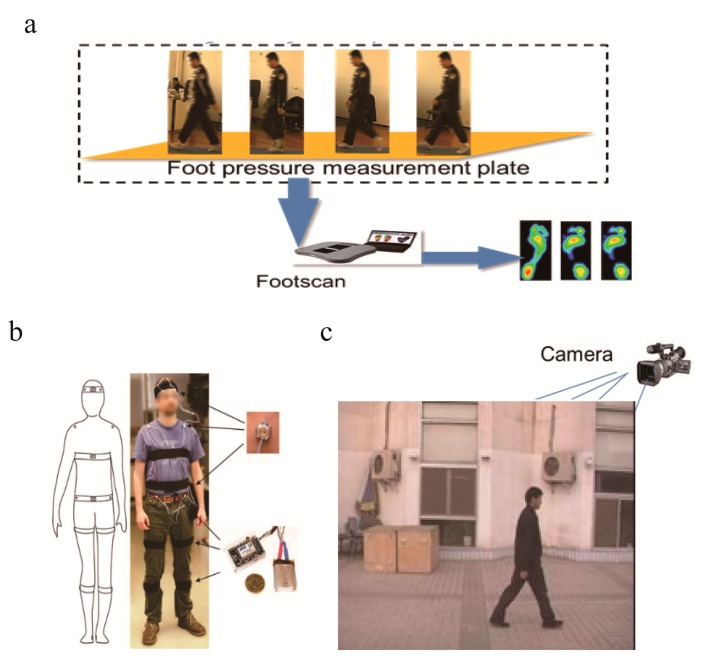
Some examples of sensor-based gait information acquisition systems. (**a**) Tactile sensor-based approach [23]; (**b**) Wearable sensor-based approach [24]: schematic (left) and photograph (right); (**c**) Video sensor-based approach [25].

Over the past ten years, gait recognition, which utilizes the manner of walking to identify individuals, has obtained extensive interest in the communities of biometric recognition and video surveillance [1–9]. Compared to other biometrics, such as face [10], fingerprint [11], palmprint [12], iris [13], DNA [14], or a combination of these traits [15,16], gait offers the potential for recognition at a distance or at low resolution and can be applied inconspicuously. Depending on the sensors used, a gait recognition system can be classified into three groups, which are tactile sensor-based, wearable sensor-based and video sensor-based. Tactile sensors generally refer to the multi-degree-of-freedom pressure sensor [17,18]. These tactile sensors are usually placed along a particular floor to collect the pressure signal generated when people walk across it. Figure 1a shows an example of a tactile sensor-based approach. Wearable sensors [19] are attached or worn on the key points of different body parts, such as the waist, pockets, shoes and so forth (see Figure 1b), in order to collect the speed, acceleration, position and other information about human gait. Although the sensors can directly access the motion information of specified parts and obtain accuracy data, they require complex equipment in collection and most applications of these methods are limited in medical research. In contrast to tactile sensor-based and wearable sensor-based gait recognition, this survey focuses on the most widely used video sensor-based gait recognition [6,20–22]. The video sensor-based system typically consists of several digital or analog cameras with suitable optics for acquiring the gait data from a distance (see Figure 1c).

**Figure 2.**
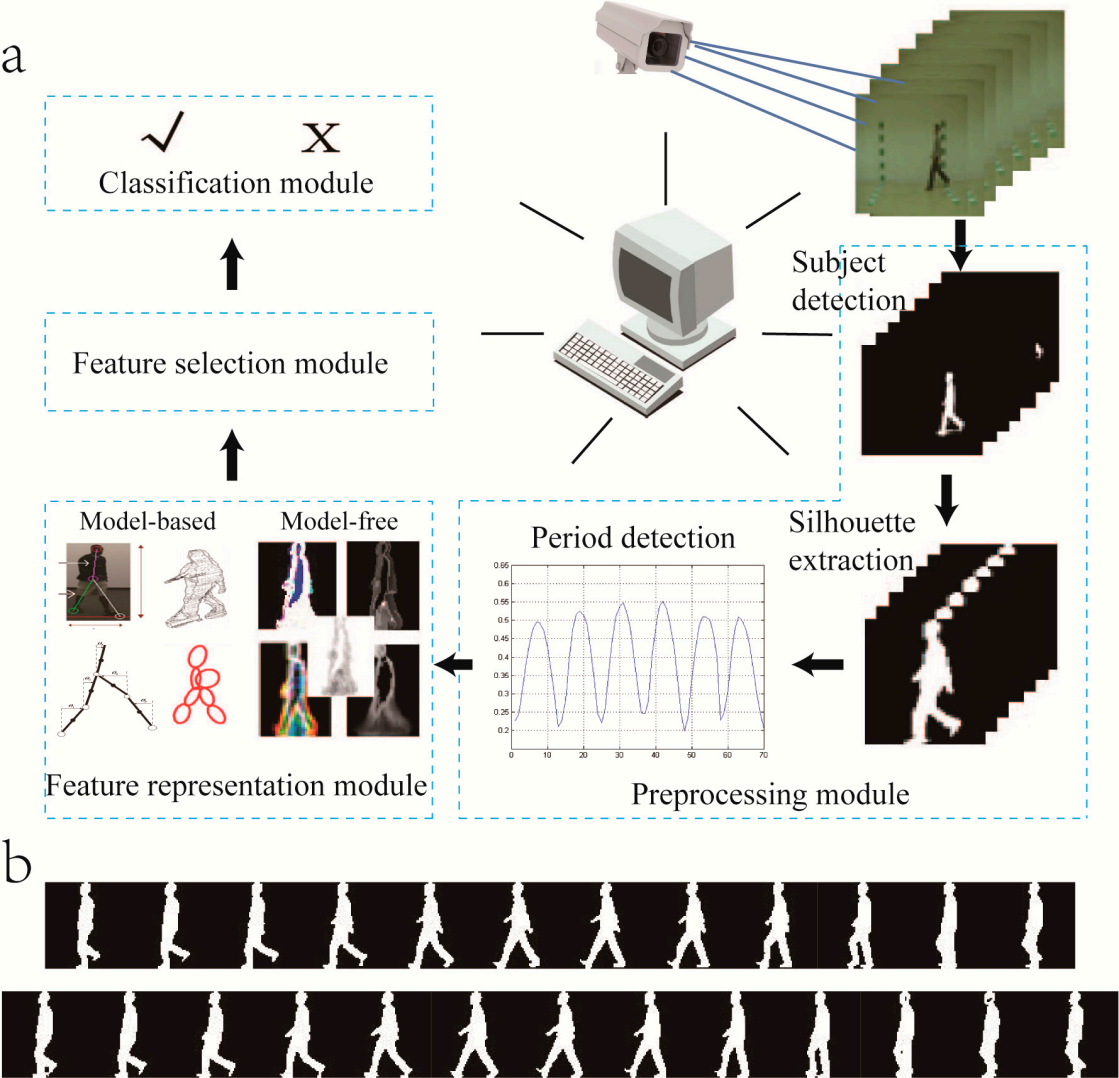
(**a**) The general framework of a video sensor-based gait recognition system. The camera-based sensor captures gait information and sends the data to computers. The system includes four modules, which are the preprocessing module (*i.e.*, subject detection and silhouette extraction from the original video), feature representation module, feature selection module and classification module. Note that the model-based gait recognition may not need the preprocessing module; (**b**) The silhouette images are the results of period detection corresponding to the preprocessing module in Figure 2a.

**Figure 3.**
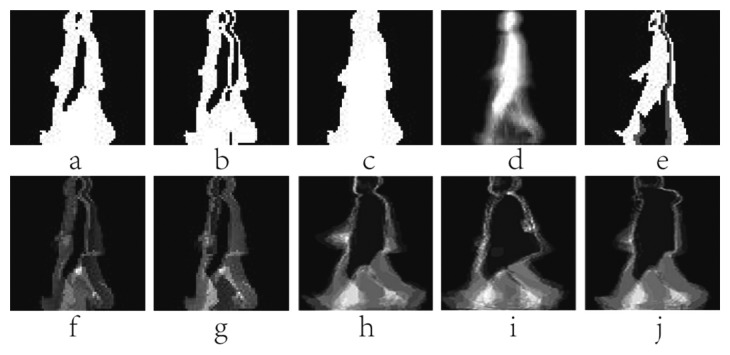
(**a**) A sample of Motion Energy Image (MEI); (**b**) A sample of Motion History Image (MHI); (**c**) An example of Motion Silhouettes Image (MSI); (**d**) An example of Gait Energy Image (GEI); (**e**) An example of Gait History Image (GHI); (**f**) The forward Single-step History Image (fSHI) sample; (**g**) The backward Single-step History Image (bSHI) sample; (**h**) The Active Energy Image (AEI) sample in normal state; (**i**) The AEI sample walking with bag; (**j**) The AEI sample walking on coat.

**Figure 4.**
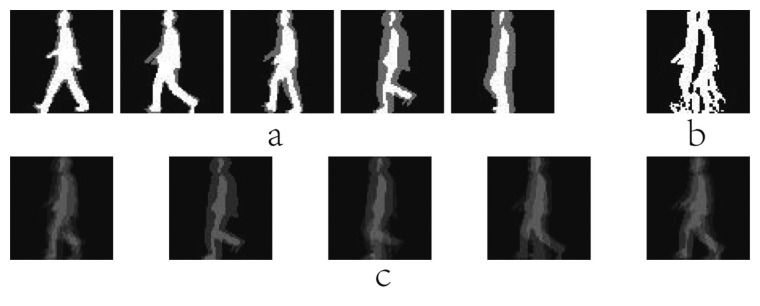
(**a**) Some samples of GMI; (**b**) The sample of GDI; (**c**) Some MGEI samples.

**Figure 5.**
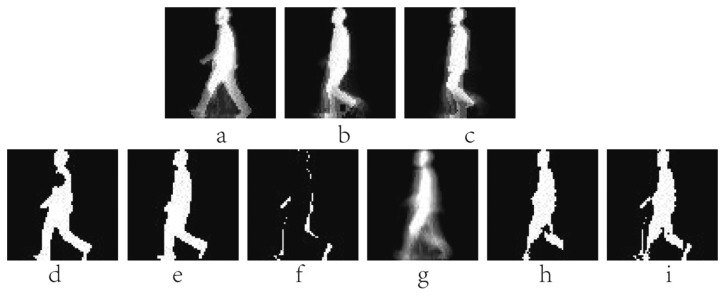
(**a**) An MIEI samples for n = 6 in Equation (11); (**b**) An MIEI samples for *n* = 6 in Equation (11); (**c**) An MIEI samples for *n* = 6 in Equation (11); (**d**) An incomplete silhouette at *t*−1; (**e**) The silhouette at *t*; (**f**) The positive portion of the frame difference; (**g**) The GEI; (**h**) The DEI; (**i**) The FDEI of (d).

**Figure 6.**
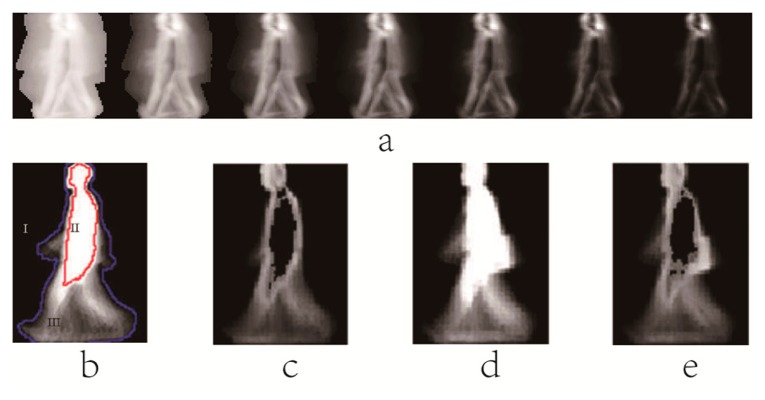
(**a**) Some samples of gamma corrected DWMs [70] (from left to right is *γ* = 0.1, 0.3, 0.5, 0.7, 1, 1.5, 2); (**b**) The GEI in normal state; (**c**) The EGEI in normal state; (**d**) The GEI walking with bag; (**e**) The EGEI walking with bag.

**Figure 7.**
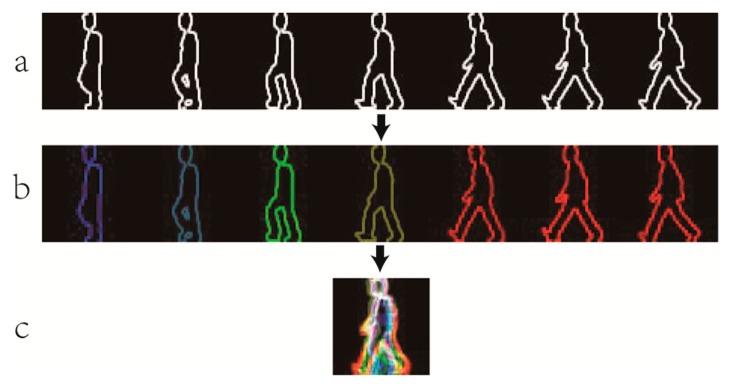
An example of generating a CGI template. (**a**) The contour images; (**b**) The multi-channel contour images; (**c**) A CGI template of a gait period.

**Figure 8.**
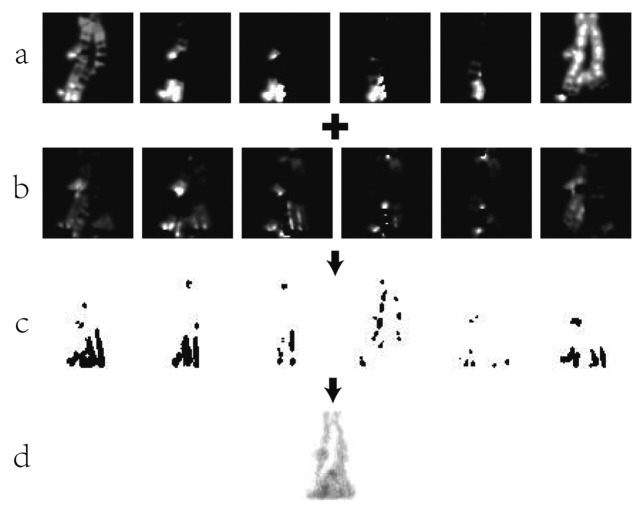
Optical flow silhouette images. (**a**) Horizontal optical flow field images; (**b**) Vertical optical flow field images; (**c**) The magnitude of optical flow fields' images; (**d**) The binary flow images.

**Figure 9.**
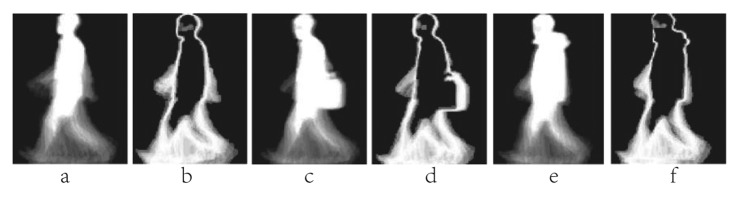
Some GEI and GEnI samples. (**a**) The GEI in normal state; (**b**) The GEnI in normal state; (**c**) The GEI walking with bag; (**d**) The GEnI walking with bag; (**e**) The GEI walking in a coat; (**f**) The GEnI walking in a coat.

**Figure 10.**
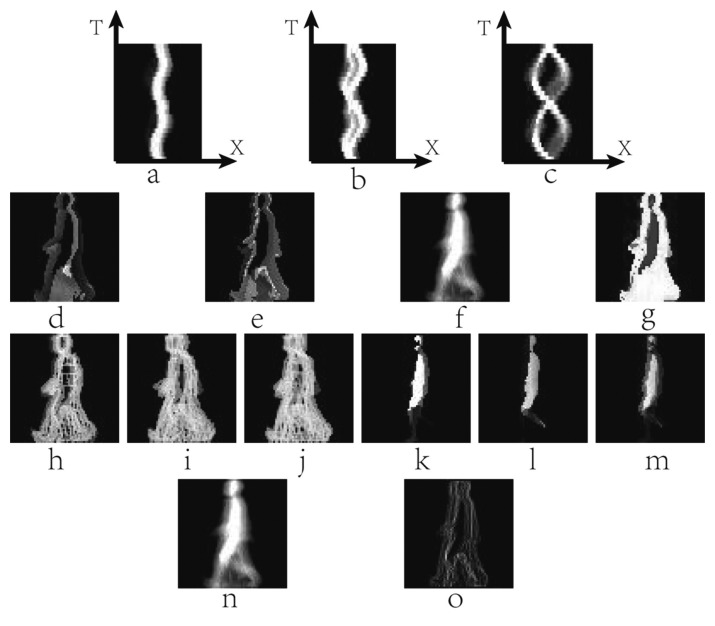
(**a**) The upper X-T PEI of a gait sequence; (**b**) The middle X-T PEI of a gait sequence; (**c**) The lower X-T PEI of a gait sequence; (**d**) The fSHI of channel R; (**e**) The fSHI of channel G; (**f**) The GEI of channel B; (**g**) The CGHI; (**h**) and (**i**) are MSCTs of a gait period; (**j**) The MSCT of a silhouette sequence; (**k**) and (**l**) are SSTs of a gait period. (**m**) The SST of a silhouette sequence; (**n**) An example of AME; (**o**) An example of MMS.

The general framework of the video sensor-based gait recognition system includes four modules [21], which are preprocessing module [26–30] (*i.e.*, subject detection and silhouette extraction from the original video), feature representation module [4,31–39], feature selection module [35,40,41] and classification module [42–44]. The framework of the video sensor-based gait recognition system is shown in Figure 2a. The camera-based sensor captures gait information and sends the data to computers. Figures 3, 4, 5, 6, 7, 8, 9 and 10 show templates generated from the periodic sequence in Figure 2b. This survey mainly focuses on the feature representation module.

There are various approaches available for video sensor-based human gait representation, which can be roughly divided into two broad categories: the model-based and model-free approaches [5,45]. The model-based approach aims to explicitly model human body or motion according to prior knowledge. Generally, each frame of a walking sequence is fitted to the model of the human body and the parameters, such as motion trajectories [46], joint angles [5], hip position [47], limb lengths [48], body part ellipses [49] and physical distances [50], gathered from moving bodies are measured on the model as gait features for recognition. One such approach represents a gait silhouette as seven regions of ellipses. Then, the ellipses' parameters are computed as gait features for recognition [49]. Another method utilizes the pendulum model to guide the motion extraction process [51]. Recently, Zeng *et al.* [5] proposed employing the lower limb joint angles to characterize gait features. Some researchers studied the motion trajectories or joints under multi-cameras conditions [20,52,53].

Model-based approaches are insensitive to background cluttering and noise. These methods are easy to be understood, and generally view and scale invariant. However, model-based approaches suffer from several drawbacks. First, it is difficult to accurately locate the joints' position due to the highly flexible structure of non-rigid human body and to self-occlusion [54,55]. Second, model-based approaches are sensitive to the quality of gait sequences. Third, and the greatest disadvantage of the model-based approaches is their large computation cost and relatively high time costs due to parameters calculations, complex feature extraction and matching in these methods. Thus, it is somewhat difficult for the model-based approach to be applied in real environment. Therefore, current literature focuses more on model-free approaches.

Model-free approaches [4,31–39,56] aim to utilize the motion information directly to identify individuals and does not need the prior knowledge of the gait model. They usually extract statistical features from the whole silhouette without assuming the underlying structure [57]. Model-free approaches can be divided into two groups, namely temporal comparison and summary of spatio-temporal information. The temporal comparison approaches directly compare and match spatial features temporally, on a frame by frame basis. In this case, typical methods include correlation between frames and Hidden Markov Models [58]. Sarkar *et al.* [59] propose a baseline gait recognition algorithm for computing the correlation between frames by using the ratio of the intersection to the union between the probe and gallery frames. Kale *et al.* [58] employ Hidden Markov Models to distinguish between temporal data. The approach computes the likelihood generated from the Hidden Markov Model corresponding to a particular person to perform identification.

In order to reduce the challenges of comparing images on a frame by frame basis, it is more efficient to use summarized motion features spatiotemporally. A number of spatio-temporal motion summary approaches usually superpose sequences of binary silhouettes depending on certain rules. Then, the original video sensor-based silhouette sequences are transformed into a single image template for recognition. In this paper, the template is called Class Energy Image. The method generating the template is called the Class Energy Image approach. The Class Energy Image approaches characterize the gait under multiple conditions without considering the body structure and computing accurate parameters of body parts in gait recognition. The advantages of the video sensor-based Class Energy Image approach can be summarized as follows: (a) It is well suitable for real time systems because it is easy to extract the feature and computational complexity is low [21,60]; (b) It is insensitive to the quality of silhouettes comparing to model-based approaches; (c) It holds several key features of human gait including motion frequency, temporal and spatial changes of human body, and global body shape statistic [3]; (d) It is robust to silhouette errors and image noise [61]. Due to the above merits, the Class Energy Image approaches have been widely used in the state-of-art gait recognition systems [3,61–67]. Consequently, we focus on providing a comprehensive review of the past and present Class Energy Image approaches for video sensor-based gait recognition in this paper.

The organization of this paper is as follows: Section 2 provides a panoramic summary and analysis of related work in the general area of Class Energy Images. In Section 3, we evaluated and discussed the performances of various Class Energy Image approaches by experiments. The results demonstrated that some Class Energy Image approaches could attain higher recognition accuracy with good robustness and efficiency. Section 4 outlines important observations and provides promising future directions for the field.

## 2. The Class Energy Image Approach

Based on the different ways of feature extraction and Class Energy Image generation, we divided the present Class Energy Image approach for video sensor-based gait recognition into three categories: the gait information accumulation approach, gait information introduction approach and gait information fusion approach.

### 2.1. Gait Information Accumulation Approach

The gait information accumulation approach makes an original video sensor-based gait silhouette sequence represent as one or several matrix-like second-order images by using mathematical methods of average, difference, maximum and minimum operation, *etc.* The gait information accumulation approach is insensitive to incidental silhouette errors, and performs better and provides richer information than the original binary gait image. The common gait information accumulation approaches include Motion Energy Image (MEI) [33], Motion History Image (MHI) [33,39], Motion Silhouettes Image (MSI) [68], Gait Energy Image (GEI) [64,69], Averaged Silhouette (AS) [4], Gait History Image (GHI) [70], forward Single-step History Image (fSHI) [71], backward Single-step History Image (bSHI) [71], Active Energy Image (AEI) [72–74], Gait Moment Image (GMI) [75], Moment Gait Energy Image (MGEI) and Gait Deviation Image (GDI) [76], *etc.*

In 2001, Bobick *et al.* [33] transformed silhouette image sequences to Motion Energy Image (MEI) and Motion History Image (MHI). Given a preprocessed binary gait silhouette sequence *B*(*x*, *y*, *n*), MEI and MHI are defined as follows:

(1) $$\begin{array}{l} E_{\text{MEI}}(x,y,n)=\cup_{i=0}^{\tau -1}D(x,y,n-i)\\ D(x,y,n)=B(x,y,n+1)-B(x,y,n)\\ E_{\text{MHI}}(x,y,n)=\{\begin{array}{ll} \tau & \text{if}\;D(x,y,n)=1\\ \max(0,E_{\text{MHI}}(x,y,n-1)-1) & \text{oterwise} \end{array} \end{array}$$

where *n* is the frame number (moment of time) of a silhouette sequence, *x* and *y* are values in the 2D image coordinate. *τ* is the duration of a current silhouette in the sequence. The pixel value of *B*(*x*, *y*, *n*) ranges within [0,1]. *D*(*x*, *y*, *n*) is the binary difference silhouette image, which indicates regions of motion. For example, *D*(*x*, *y*, *n*) = 1 represents a motion occurrence in the *n* th frame (time) on the coordinate point (*x*, *y*). MEI accumulates all regions of motion in a gait sequence. MHI is a gray image with temporal information. The value of MHI is associated with the current moment. MEI and MHI have less computational complexity, but the static information is not sufficient. Examples of MEI and MHI are shown in Figure 3a,b. The two templates are the basis of the later research on the behaviors and gait recognition.

Lam *et al.* [68] employed Motion Silhouettes Image (MSI) to characterize a motion image sequence. MSI is generated in nearly the same way as MHI. Pixel intensity of MSI is a function of the temporal history of motion at this point, and MHI is generated by using the following algorithm:

(2) $$E_{\text{MSI}}(x,y,n)=\{\begin{array}{ll} 255 & if\;B(x,y,n)=1\\ \max(0,E_{\text{MSI}}(x,y,n-1)-1) & \text{otherwise} \end{array}$$

MSI, which is simpler than MHI, is a gray image with temporal information. The pixel value shows the motion history at this pixel. Figure 3c shows an example of MSI.

Gait identification is a special case of behavior recognition. In 2004, Han *et al.* [69] proposed Gait Energy Image (GEI). The grey-level GEI can be created from averaging the silhouettes with Equation (3).

(3) $$E_{\text{GEI}}=\frac{1}{N}\overset{N}{\underset{n=1}{\text{∑}}}B(x,y,n)$$

where *N* denotes the number of the binary silhouette images in a gait cycle. GEI reflects temporal length of each posture in a complete gait period. A pixel with higher intensity value in GEI means that human walking occurs more frequently at this position. GEI preserves the dynamic and static (shape) information of a gait sequence. The common static information is the proportion of the human body, clothing and bags, *etc.* Furthermore, there is no consideration of the time that normalizes each silhouette. Figure 3d presents an example of MSI.

Liu *et al.* [4] used average silhouette to characterize gait features. Suppose a sequence of silhouettes, S={*S*(1),⋯,*S*(*M*)}, the *i* th average silhouette (AS) in a gait cycle was expressed as

(4) $$E_{\text{AS}}(i)=\frac{1}{N_{\text{Gait}}}\overset{(i+1)N_{\text{Gait}}-1}{\underset{k=iN_{\text{Gait}}}{\text{∑}}}S(k)$$

*N_Gait_* denotes the numbers of silhouettes in a gait cycle. It is noteworthy that the theory of AS and GEI is similar.

Inspired by MHI, Liu *et al.* [70] adopted Gait History Image (GHI) to characterize the motion image sequence. GHI is obtained as follows:

(5) $$E_{\text{GHI}}(x,y)=\{\begin{array}{ll} P & if\overset{P}{\underset{n=1}{\cap }}B(x,y,n)=1\\ \sum_{n=1}^{p}D(x,y,n)\cdot (n-1) & \text{otherwise} \end{array}$$

where *P* is the number of the frames in a quarter cycle of a silhouette image sequence, and GHI is generated from 1/4 gait period. The pixel values in GHI represent the temporal changes. GHI not only contains the dynamic and static information, but also inherits the characteristics of MHI which can reflect temporal variation. However, GHI computed from a quarter gait period, it will lose amount of useful information. Figure 3e shows a GHI sample of one person.

Based on the idea of MHI in behavior recognition, Chen *et al.* [71] proposed a Single-step History Image (SHI). The forward and backward difference image between two adjacent silhouettes can obtain forward Single-step History Image (fSHI) and backward Single-step History Image (bSHI), respectively. fSHI and bSHI are defined as follows:

(6) $$\begin{array}{llll} E_{\text{fSHI}}(x,y) & =\overset{N_{\lambda }-1}{\underset{n=1}{\text{∑}}}E_{\text{fSHI}}(x,y,n), & E_{\text{fSHI}}(x,y,n) & =\{\begin{array}{cc} n\tau_{1} & \text{if}\;D(x,y,n)=1\\ 0 & \text{otherwise} \end{array}\\ E_{\text{bSHI}}(x,y) & =\overset{N_{\lambda }-1}{\underset{n=1}{\text{∑}}}E_{\text{fSHI}}(x,y,n), & E_{\text{bSHI}}(x,y,n) & =\{\begin{array}{cc} n\tau_{1} & \text{if}\;D(x,y,n)=-1\\ 0 & \text{otherwise} \end{array} \end{array}$$

where *N_λ_* is the number of the frames in the *λ* th (*λ*=1,2) single-step period. *τ*_1_ is the gray scale difference, and the initial value of *τ*_1_ is 255/(*N_λ_* − 1) in a 8-bit gray image. fSHI and bSHI reveal emerging and disappear silhouette areas over time, respectively. The two templates can describe the silhouette changes between frames, and reflect the time-spatial information of gait. Examples of fSHI and bSHI are shown in Figure 3f,g.

GEI is easily influenced by clothing and carrying conditions, meanwhile it neglects some dynamic information. In order to address these problems, Zhang *et al.* [74] presented Active Energy Image (AEI), AEI is obtained as follows:

(7) $$\begin{array}{l} E_{\text{AEI}}=\frac{1}{N}\overset{N-1}{\underset{n=1}{\text{∑}}}D_{n}(x,y,n)\\ D_{n}(x,y,n)=|B(x,y,n+1)-B(x,y,n)| \end{array}$$

AEI aims at extracting the active regions by calculating the difference between two adjacent silhouettes in the gait sequence. AEI contains more temporal characteristics for discriminant than GEI. Moreover, AEI can reduce the influence of carrying conditions. When clothing and loading change little between adjacent silhouettes, the influence could be ignored. However, AEI only makes use of the dynamic parts in the silhouette images without consideration of the static information. Figure 3h–j shows some AEI samples of a subject under normal walking, walking with bag and walking on coat conditions.

Each cycle only includes one GEI or GHI template, which easily leads to the problem of insufficient training samples. To address this problem, Ma *et al.* [75] utilized Gait Moment Image (GMI) to express a silhouette sequence. GMI is the gait probability image at each key moment in all gait cycles. The corresponding gait images at a key moment are averaged as GEI of this key moment. GMI at the *k* th key moment is calculated as:

(8) $$E_{\text{GMI}}(x,y,k)=\frac{1}{C}\overset{C}{\underset{i=1}{\text{∑}}}B_{i}(x,y,k)$$

where *C* is the number of the gait cycles in a gait sequence. Figure 4a shows five GMIs of the same person. Ma *et al.* [76] further improved the image quality of each key frame, and uniformly choose *S* interested moments as the key frames in a gait cycle. Moment Gait Energy Image (MGEI) at the *k* th key moment can be given as follows:

(9) $$E_{M\text{GEI}}(x,y,k)=\frac{1}{2\times \sum_{i=1}^{\overset{S}{\;}/\underset{2}{\;}}r^{i}}\sum_{i=1}^{\overset{S}{\;}/\underset{2}{\;}}\sum_{i=-1,1}{r_{1}}^{i}\times \frac{1}{C}\sum_{C=1}^{C}B(x,y,(k+S+i\times i)\%S)$$

where *r*_1_ is the decline coefficient. Smaller values of *r*_1_ would make MGEI quite similar to *B*(*x*,*y*,*k*). While bigger values of *r*_1_ would make each frame lose uniqueness. Some key frames are sufficiently employed to create the MGEI, and the way of its calculation is greatly different from GEI. Furthermore, MGEI has temporal information between frames. Some examples of MGEI are shown in Figure 4c. Furthermore, Gait Deviation Image (GDI), which represented a kind of accumulation of the deviations between original silhouette images and the moment probability images, is obtained as follows:

(10) $$\begin{array}{l} E_{\text{GDI}}(x,y,k)=\{\begin{array}{cc} \max(E_{\text{GDI}}(x,y,k-1)-255/N,0) & \text{if}\;E_{\text{DGMI}}(x,y,k)=0\\ \max(E_{\text{GDI}}(x,y,k-1),E_{\text{DGMI}}(x,y,k)) & \text{else} \end{array}\\ E_{\text{DGMI}}(x,y,k)=\frac{1}{C}\overset{C}{\underset{i=1}{\text{∑}}}{|B}_{i}(x,y,k)-E_{G}(x,y,k)| \end{array}$$

where *N* is the gait period in a gait sequence. *E_G_*(*x*,*y*,*k*) is the *k* th key moment of GMI or MGEI. The result of the Equation (10) at the final key moment is GDI. Figure 4b shows the examples of GDI generated from GMI. Compared to GEI, GDI reflects more dynamic information, but less static information.

The disadvantages of the Class Energy Image based on the key frames are: it is not easy for GMI or MGEI to select key moments from cycles with different periods.

The gait information accumulation approach is an effective representation of a video sensor-based gait sequence, which not only saves storage space and computational time, but also attains better recognition performance. The disadvantage is that some useful information may be lost, and the problem of inadequate training samples is raised. Table 1 characterizes the expressions, motion information (*i.e.*, dynamic, static and temporal information) and computational complexity of the gait information accumulation approach. The temporal information refers to the fore-and-after relations in the gait feature description. For example, the GEI contains no temporal information. The reason for this phenomenon is that GEI representation is the same as that of the original sequence when the frames in a gait sequence are disordered. Real-time gait recognition systems requires low computational complexity [1,77]. The Class Energy Images of gait information accumulation approach have the consistent computational complexity, which reveals good performances of real-time.

### 2.2. Gait Information Introduction Approach

Gait information accumulation approach only reformulates a video sensor-based gait sequence by using templates as holistic feature and arguably loses some intrinsic dynamic characteristics of the gait pattern. In order to weaken this effect, gait information introduction approach introduces dynamic information to the static silhouette images based on GEI by adopting the mathematical transformation methods of average, difference and motion regions extraction, *etc.* The general gait information introduction approaches include: Motion Information Energy Image (MIEI) [78], Frame Difference Energy Image (FDEI) [79–81], Enhance Gait Energy Image (EGEI) [55,72,82], Chrono Gait Image (CGI) [83–85], Gait Flow Image (GFI) [86,87], and Gait Entropy Image (GenI) [56,88], *etc.*

Masoud *et al.* [78] applied the weighted average idea to GEI, and Motion Information Energy Image (MIEI) was devised. MIEI was started with a weighted average at time (frame) *n*. MIEI is computed as follows:

(11) E(x,y,n)=α×B(x,y,n−1)+(1−α)×E(x,y,n−1)

where *α* is a parameter within [0,1]. *E*(*x*, *y*, *n*) can be updated consecutively. The recent image makes a greater contribution to *E*(*x*, *y*, *n*). More dynamic discriminative information is introduced to GEI by selecting appropriate *E*(*x*, *y*, *n*−1) and *α*. However, the values of *E*(*x*, *y*, *n*−1) and *α* vary from different subjects. Some examples of MIEI are shown in Figure 5a–c.

To suppress the influence of silhouette incompleteness for identification, Chen *et al.* [79] proposed Frame Difference Energy Image (FDEI). A gait cycle is divided into clusters and the dominant energy image (DEI) is obtained by denoising the averaged image of each cluster. The frame difference is calculated by subtracting two consecutive frames. FDEI representation is constructed as the summation of its corresponding cluster's DEI and the positive portion of its frame difference. FDEI is defined as follows:

(12) $$\begin{array}{l} E_{\text{FDEI}}(x,y)=F(x,y,n)+E_{\text{DEI}}^{c}(x,y)\\ {E^{c}}_{\text{DEI}}(x,y)=\{\begin{array}{ll} \frac{1}{N_{c}}\underset{n\in N_{C}}{\text{∑}}B(x,y,n) & \text{if}\;\frac{1}{N_{c}}\underset{n\in N_{C}}{\text{∑}}B(x,y,n)\geq T\\ 0 & \text{otherwise} \end{array}\\ F(x,y,n)=\{\begin{array}{ll} 0 & B(x,y,n)\geq B(x,y,n-1)\\ B(x,y,n-1)-B(x,y,n) & \text{otherwise} \end{array} \end{array}$$

where *N_C_* is the number of frames in the *C* th cluster, and it represents the time (frame) set of silhouettes. Average distortion is used to choose the cluster number [79], which decreases with the increased cluster number. If the average distortion does not change appreciably beyond certain number, it can be chosen as the cluster number. When the value of cluster number is very small, some useful information for identification may miss. The larger cluster number leads to great computation complexity, but has little improvement on the recognition performance. The threshold *T* varies with different periods or subjects, depending on the quality of the silhouettes. *B*(*x*, *y*, 0) is viewed as the last frame in a period. When *B*(*x*, *y*, *n*) is incomplete and *B*(*x*, *y*, *n*−1) is complete, the missing portions of the frame are contained in *F*(*x*, *y*, *n*). When the *B*(*x*, *y*, *n*) and *B*(*x*, *y*, *n*−1) are both incomplete, the missing portions can be compensated by *E^c^_DEI_*(*x*,*y*). FDEI representation helps to suppress the influence of the missing portions and preserve the characteristics of *B*(*x*, *y*, *n*). FDEI is robust to incomplete silhouette images. Moreover, FDEI embodies both static and kinetic information between frames. Figure 5d–i demonstrate some images during the construction of FDEI, where Figure 5d,e show silhouettes *B*(*x*, *y*, *n*−1) and *B*(*x*, *y*, *n*), respectively. Figure 5f shows the movement portion of *B*(*x*, *y*, *n*). A GEI of the cluster is shown in Figure 5g. Figure 5h embodies the dominant energy of GEI, which is obtained by denoising Figure 5g. Figure 5i is FDEI of *B*(*x*, *y*, *n*). Comparing (i) with Figure 5d, it can be seen that FDEI contains the movement portion and partially compensates the incompleteness of *B*(*x*, *y*, *n*).

Yang *et al.* [55] also devised an Enhance Gait Energy Image (EGEI) representation method. The method applied dynamic region analysis to improve dynamic information of GEI. Then a better performance can be attained than the conventional GEI method. The intensity in GEI indirectly reflects the time spent at each stance: the regions with high intensity and low intensity marked with “I” and “II” in Figure 6b are essentially the same among different individuals. While the dynamic region marked with “III” in Figure 6b, which is the area between the red and the blue circle, embodies the swing of limbs and the inclination of head and torso. In this region, different people have different distributions of pixel intensity values. Identity can be differentiated by these individual characteristics. The dynamic region in GEI is enhanced by a pixel-wise multiplication with DWM by the following equation:

(13) $$\begin{array}{ll} E_{\text{EGEI}}(x,y) & =E_{\text{GEI}}(x,y)\times {\left(T_{\text{DWN}}(x,y)\right)}^{\gamma }\\ \sigma_{\text{GEI}}(x,y) & =\sqrt{\frac{1}{A}{\overset{A}{\underset{i=1}{\text{∑}}}\;\left[\frac{1}{N}\overset{N}{\underset{m=1}{\text{∑}}}G_{m}^{i}(x,y)-\frac{1}{A}\overset{A}{\underset{i=1}{\text{∑}}}\frac{1}{N}\overset{N}{\underset{m=1}{\text{∑}}}G_{m}^{i}(x,y)\right]}^{2}} \end{array}$$

where *A* is the total number of classes in the training set. *N* is the number of samples in each class. The total number of the samples in the training set is *M* = *NA*. $G_{m}^{i}=\left(x,y\right)$ denotes the *m* th GEI from the *i* th class. *σ_GEI_*(*x*,*y*) is the standard deviation which reflects the variance between different classes. *T_DWN_*(·) is the dynamic weight mask (DWM), which is the *σ_GEI_*(*x*,*y*) normalized to [0,1] and indicates the degree of dynamics in (*x*,*y*). *γ* is a gamma correction tuning parameter, and Figure 6a demonstrates a group of gamma corrected DWMs with different *γ* values. However, EGEI is still heavily affected by other factors such as clothing and carrying object. Some GEI and EGEI samples of an individual extracted from the gait sequences collected under normal walking, walking with bag conditions are shown in Figure 6b–e.

Temporal information of GEI may be lost, whereas it reduces the effect of the noises. In order to well preserve the temporal information of gait patterns, Wang *et al.* [83] put forward a multi-channel temporal encoding technique, named as Chrono Gait Image (CGI), to encode a gait sequence to a multi-channel image. CGI is defined as follows:

(14) $$\begin{array}{l} E_{\text{CGI}}(x,y)=\frac{1}{p}\begin{array}{cc} \sum_{i=1}^{p}\sum_{t=1}^{n_{i}}C_{t}(x,y) & C_{t}(x,y)=H(x,y) \end{array}\times C_{i}(r_{t})\\ B(r_{t})=C_{1}(r_{t})=\{\begin{array}{cc} (1-2r_{t})I & 0\leq r_{t}\leq 1/2\\ 0 & 1/2\leq r_{t}\leq 1 \end{array}\\ G(r_{t})=C_{2}(r_{t})=\{\begin{array}{cc} \begin{array}{cc} 2r_{t}I & 0\leq r_{t}\leq 1/2\\ (2-2r_{t})I & 1/2\leq r_{t}\leq 1 \end{array} & r_{t}=\frac{W_{t}-W_{\min}}{W_{\max}-W_{\min}} \end{array}\\ R(r_{t})=C_{3}(r_{t})=\{\begin{array}{cc} 0 & 0\leq r_{t}\leq 1/2\\ (2r_{t}-1)I & 1/2\leq r_{t}\leq 1 \end{array} \end{array}$$

where *p* is the number of silhouettes in the 1/4 gait period. *H*(*x*,*y*) is the gait contour information which can be got by local information entropy method. *C_i_*(*r_t_*) is the *t* th frame different weights in different channels depending on their position in the 1/4 gait periodic sequence. *C_t_*(*x*,*y*) indicates the multi-channel contour image. *n_i_* is the number of the *C_t_*(*x*,*y*) in the *i* th 1/4 gait period. *W_t_* expresses the average width of the leg region in the *t* th frame, *W*_min_ and *W*_max_ are the extreme widths of the 1/4 period which the *t* th frame belongs to. Figure 7 represents the process of generating CGI. However, CGI may lose some dynamic information, such as the frequency information. The accuracy of CGI depends on the number of 1/4 gait period in a silhouette sequence.

Optical flow is a mode of target motion in scenes. Optical flow has been well used in the field of moving target detection, segmentation and tracking. Toby *et al.* [86] introduced optical flow to gait representation, and presented Gait Flow Image (GFI), which is obtained as in Equation (15).

(15) $$\begin{array}{l} E_{\text{GFI}}(x,y)=\frac{\sum_{n=1}^{N-1}F_{n}(x,y)}{N}\\ F_{i}(x,y)=\{\begin{array}{ll} 0 & \text{if}\;\text{Mag}F_{i}(x,y)\geq 1\\ 1 & \text{otherwise} \end{array}\\ \text{Mag}F_{i}(x,y)=\sqrt{{\left(\mu F_{i}(x,y)\right)}^{2}+{\left(\nu F_{i}(x,y)\right)}^{2}} \end{array}$$

where *uF_i_*(*x*,*y*) and *vF_i_*(*x*,*y*) are the horizontal and vertical optical flow field. *MagF_i_*(*x*,*y*) is the resultant magnitude of *uF_i_*(*x*,*y*) and *vF_i_*(*x*,*y*). *F_i_*(*x*,*y*) is a binary flow image. Figure 8 demonstrates an example of generating GFI. GFI represents the motion of silhouette images. The dark region in Figure 8c,d means the region with movement, and the white region means the region without any movement. More dynamic information can be embodied when introducing optical flow to gait representation. However, GFI will introduce the information unrelated with identification when image sequences have low quality.

GEI is sensitive to static condition variation, such as clothing, carrying condition (backpack, briefcase, handbag, *etc.*), and shoe-wear. Bashir *et al.* [56] proposed the Gait Entropy Image (GEnI) to distinguish the dynamic and static areas of GEI by measuring Shannon entropy at each pixel location in GEI. The intensity value of the silhouettes at a fixed pixel location is considered as a discrete random variable. Shannon entropy measures the uncertainty associated with the random variable over a complete gait cycle. GEnI is computed as:

(16) $$E_{\text{GEnI}}(x,y)=-E_{\text{GEI}}(x,y)\log_{2}E_{\text{GEI}}(x,y)-(1-E_{\text{GEI}}(x,y))\log_{2}(1-E_{\text{GEI}}(x,y))$$

GEnI measured the relevance of gait features extracted from GEI, and automatically selected static condition invariant features for gait recognition. Since the dynamic region has more uncertainty, the intensity values of GEnI are larger in the dynamic region and smaller in the static region. Figure 9 shows some GEI and GEnI samples of one person walking normally, walking with bag and walking while wearing a coat, where (a, c, e) are the GEI samples, and (b, d, f) are the corresponding GEnI ones, respectively. Dynamic and static information could be easily distinguished by constructing the Shannon entropy in GEI.

Gait information introduction approach can highlight the dynamic information, meanwhile preserving the static information. Table 2 characterizes the expressions, motion information and computational complexity of the gait information introduction approach. This kind of approach is insensitive to noises, and not critical to the identification method. In addition, the computational complexity of Class Energy Images in this section has significant differences. Especially, it is obvious that CGI and GFI have extremely higher computational complexity than others.

### 2.3. Gait Information Fusion Approach

Gait information fusion approach employs feature layer fusion and decision layer fusion method [89] to achieve the fusion of static, dynamic and temporal information. Gait information fusion method usually includes X-T Plane Energy Image (X-T PEI) [90], Color Gait History Image (CGHI) [91], Motion Silhouette Contour Template (MSCT), Static Silhouette Template (SST) [92,93], Mean Motion Shape (MMS) and Average Motion Energy (AME) [94], *etc.*

Guo *et al.* [90] reformulated a silhouette sequence as a third-order tensor with column, row and time modes, which was XYT form. The human body can be divided into three parts when the hip and knee are viewed as the demarcation point, and the body is mapped to the X-T plane. X-T Plane Energy Image (X-T PEI) can be generated by Equation (17).

(17) $$E_{X-T\begin{array}{c} \begin{array}{c} \text{PEI} \end{array} \end{array}}(x,n)=\frac{1}{H}\overset{H}{\underset{y=1}{\text{∑}}}B(x,y,n)$$

where *H* is the body height of the silhouette images. The three X-T PEIs, which has static and dynamic information, are fused based on series in the feature layer. The X-T PEIs of the three body parts are represented in Figure 10a–c. Moreover, X-T PEI can detect the gait period.

Further improved SHI [71], Chen [91] devised the Color Gait History Image (CGHI) to describe the temporal-spatial gait information. CGHI consists of three channels. The channels of R and G are fSHIs which views the standing on one leg and two legs as the start of a period, respectively. Moreover, the channel of B is GEI. Equation (17) gives the MATLAB expression of CGHI, where *I_R_*, *I_G_* and *I_B_* are the three channels of CGHI. Figure 10d–g show the example of generating CGHI.

(18) $$\begin{array}{l} I_{R}(x,y,1)=E_{{\text{fSHI}}_{1}}(x,y)\\ I_{G}(x,y,2)=E_{{\text{fSHI}}_{2}}(x,y)\\ I_{B}(x,y,3)=E_{\text{GEI}}(x,y) \end{array}$$

Unlike the aforementioned Class Energy Image approaches, Lam *et al.* [93] constructed two templates, the Motion Silhouette Contour Template (MSCT) and Static Silhouette Template (SST), from a sequence of silhouette images for recognition. MSCT and SST embed critical spatial and temporal information, and they are defined as follows:

(19) $$\begin{array}{l} E_{\text{MSCT}}(x,y,n)=\{\begin{array}{cc} 255 & \text{if}\;A_{i}(x,y,n)=1\\ \max(0,A_{i}(x,y,n)-\frac{255}{P}) & \text{otherwise} \end{array}\\ A_{i}(x,y,n)=B_{i}(x,y,n)-\underset{s\in S}{\cap }{\left(B_{i}(x,y,n)\right)}_{-s}\\ E_{\text{SST}}(x,y,n)=\{\begin{array}{ll} 1 & \text{if}\;\begin{array}{cc} E_{\text{SST}}(x,y,n)= & E_{\text{SST}}(x,y,n-1) \end{array}\\ 0 & \text{otherwise} \end{array} \end{array}$$

where *P* is the number of frames in a gait period. $\underset{s\in S}{\text{∩}}{\left(B_{i}(x,y,n)\right)}_{-s}$ is the eroded silhouettes. *S* is the structuring element. MSCT contains information about the movement characteristics of a human gait and SST embeds information about the static characteristics of a human gait. These templates are used together for gait recognition. Figure 10h–m demonstrate some MSCT and SST samples of a silhouette sequence with two gait periods, where Figure 10h,i are MSCT samples of a gait period, and Figure 10k,l are the corresponding SST ones, respectively. Figure 10j,m are MSCT and SST of the silhouette sequence, respectively. However, the method is affected by the quality of the silhouettes. The sample category was determined by decision layer fusion strategy:

(20) $$\begin{array}{l} \text{SimScore}(u,v)={\text{SimScore}}_{\text{MSCT}}({\text{MSCT}}_{u},{\text{MSCT}}_{v})+{\text{SimScore}}_{\text{SST}}(SST_{u},SST_{v})\\ \begin{array}{cc} \min\text{SimScore}(u,i)=\text{SimScore}(u,v) & i=1,2,\ldots ,N_{\text{train}.} \end{array} \end{array}$$

where the similarity score *SimScore*(*u*,*v*) represents the level of similarity between the testing sample *u* and the training sample *v*. The *SimScore_MSCT_* and *SimScore_SST_* can be computed by Equation (21).

(21) $$\begin{array}{l} {\text{SimScore}}_{\text{Temp}}({\text{Temp}}_{u},{\text{Temp}}_{v})=\frac{\|E_{{\text{Temp}}_{u}}-E_{{\text{Temp}}_{v}}\|}{\bar{{\text{SimScore}}_{\text{Temp}}}}\\ \bar{{\text{SimScore}}_{\text{Temp}}}=\frac{\sum_{i=1}^{N_{\text{train}}}\sum_{j=1}^{N_{\text{test}}}\|E_{{\text{Temp}}_{i}}-E_{{\text{Temp}}_{j}}\|}{N_{\text{train}}*N_{\text{test}}} \end{array}$$

where Temp represents *MSCT* or *SST*. *N_train_* and *N_test_* are the numbers of training and testing samples.

Wang *et al.* [94] adopted two templates, which were Mean Motion Shape (MMS) and Average Motion Energy (AME), to describe the overall shape and the moving parts' features. The two templates can be calculated as follows:

(22) $$\begin{array}{ll} E_{\text{AME}} & =\frac{1}{N}\overset{n}{\underset{n=1}{\text{∑}}}B_{n}(x,y,n)\\ E_{\text{MMS}} & =\overset{n}{\underset{j=1}{\text{∑}}}\left(s_{j}{s_{j}}^{T}\right)/\left({s_{j}}^{T}s_{j}\right) \end{array}$$

AME is the same as GEI, which is shown in Figure 10n. MMS describes the changes of the shape contour, which is attained by utilizing the edge tracking algorithm. The normalized contour can be represented as *s*=[*u*_1_,*u*_2_,…,*u_k_*]^T^. MMS is the eigenvector that the largest eigenvalue of *E_MMS_* corresponds to. The example of MMS is demonstrated in Figure 10o. AME and MMS are used for recognizing respectively. The recognition results are fused together on decision layer.

Table 3 characterizes the expressions, motion information and computational complexity of the gait information fusion approach. Class Energy Images of gait information fusion approach have inconsistent computational complexity. In addition, gait information fusion approach mainly takes different feature images into consideration, and there is little correlation between different feature images. It is a promising direction to study the extraction of different features, and to fuse them using different fusion strategy.

## 3. Experiments and Analysis

**Figure 11.**
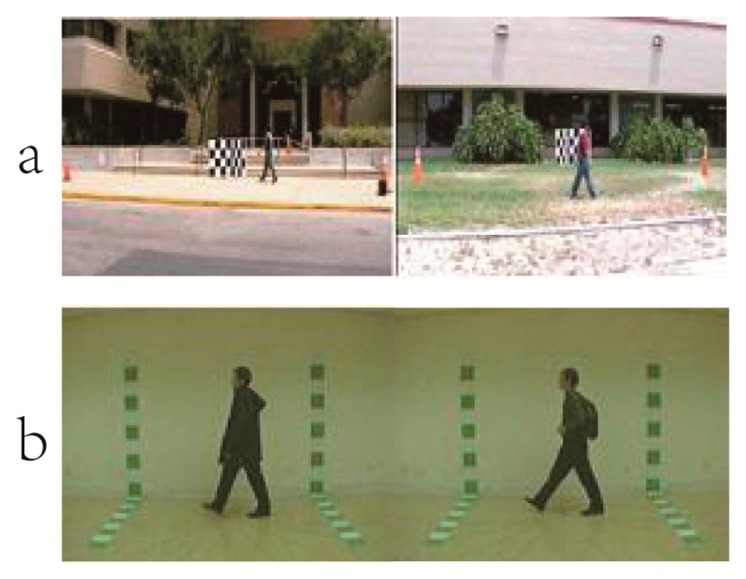
Examples of the internationally public datasets (**a**) University of South Florida (USF) HumanID dataset; (**b**) CASIA dataset B.

We evaluated the various video sensor-based Class Energy Image approaches by performing experiments on two benchmark public datasets: CASIA B dataset [61] and University of South Florida (USF) HumanID dataset [59], some video examples in the gait datasets are demonstrated in Figure 11.

All experiments are implemented by Matlab and tested on a Core 2 Duo 3.17 GHz computer with 2 GB memory.

### 3.1. Experimental Settings

The USF dataset is the outdoor gait videos which are obtained under remotely complex backgrounds. The cameras were a consumer-grade Canon Optura (for the concrete surface) and an Optura PI (for the grass surface) camera. These are progressive-scan, single-CCD cameras capturing 30 frames per second with a shutter speed of 1/250 s and with autofocus left on, as all subjects were essentially at infinity [59]. The quality of the silhouettes extracted is poor. This database consists of 122 individuals walking in elliptical paths in front of the camera. For each person, there are up to 5 covariates: viewpoints (left/right, *i.e.*, R/L), surface types (concrete/grass, *i.e.*, C/G), carrying conditions (with/without a briefcase, *i.e.*, BF/NB), shoe types (A/B) and time (T). The USF data set [59] contains 1 gallery (training) set and 12 probe (testing) sets as shown in Table 4. The gallery set contains 122 sequences. Individuals are unique in the gallery and each probe set, and there are no common sequence among the gallery set and all probe sets. Furthermore, we evaluated the various Class Energy Image approaches under the above mentioned experimental settings, which was conducive to a horizontal comparison between different Class Energy Image approaches. The noises of the silhouettes in the USF dataset were larger than that in the CASIA dataset. The experiments resulted on the USF dataset better suggested the robustness of the various Class Energy Image approaches for such noises. There are at least 5 periods for each silhouettes sequence in the USF dataset, and we determined that the start of the periods were double support positions. In order to reduce the computational complexity, we selected a gait period sequence to perform and analyze the experiments.

The CASIA B database includes 124 individuals. This video dataset was attained from USB cameras (Model: Fametech 318SC) in the indoor environment [61]. The quality of the silhouettes in the CASIA B database is high. Each individual has 3 kinds of walking state: 6 videos of normal gait, 2 videos of walking with bag and 2 videos of walking on coat, which are named nm-01 to nm-06, bg-01, bg-02, cl-01 and cl-02. Moreover, each individual has been captured from 11 different views. We only employed the 90° view to analyze the performance of the Class Energy Image approaches under different walking states in our experiments.

The aforementioned two databases provide the silhouette benchmark images after background subtraction. Only the silhouette images of USF gait Database have been preprocessed already, and the size of the silhouettes is 128 × 88 pixels. Furthermore, the silhouette preprocessing includes horizontal alignment and size normalization. The horizontal alignment is centering the upper half silhouette part with respect to its horizontal centroid. In addition, the size normalization is proportionally resizing each silhouette image so that all silhouettes have the same height. The size of the silhouette image is resized to 64 × 64 pixels in the CASIA B database. All the experiments and analysis in the paper were begun with the preprocessed silhouette image sequences.

In all the experiments, each original Class Energy Image is directly sent to nearest neighbor classifier based on Euclidean distance without using Principal Component Analysis/Linear Discriminant Analysis (PCA/LDA) to reduce the dimensions. We employ rank order statistic to evaluate the Class Energy Images. This is defined as the cumulative probability that the actual class of a test measurement is among its *k* top matches, where *k* is called the rank.

### 3.2. Recognition Performance Analysis on the USF Dataset

The USF dataset contains a number of variations which offers experimental challenges. The quality of the extracted images from videos is poor. The Class Energy Image approaches have good adaptability for low quality gait silhouette images, and their statistical properties are used to suppress the influence of the incidental silhouette errors. According to the differences between the conditions of captured videos, the probe A-L samples were divided into three groups: (I) there are small differences between gallery samples and probe samples. The static information is mainly relied on to identify; (II) The differences are between the groups (I) and (III), and the static and dynamic information have the equally important position; (III) The differences between gallery and probe samples are much greater. The shape changes for a person among the gallery and probe sets are remarkable, and the dynamic information is mainly relied on to identify. To ease our explanation, we also reported the average performance for each group by computing the ratio of correctly recognized subjects to the total number of subjects.

Tables 5, 6 and 7 are, respectively, the performances of the gait information accumulation approach, gait information introduction approach, and gait information fusion approach on the USF database.

The Class Energy Image approaches bout key frames are based on multi-period. The selection of the key frames can affect the recognition performances, which are not applicable to the gait recognition in a real-time video monitoring system. Thus, the experiments had not been performed by this kind of approaches. The recognition performances are illustrated in Table 5 for the gait information accumulation approach. It can be seen from Table 5 that (1) GEI has a higher average recognition rate for groups (I) and (II). The rank1 average performance of GEI in group (I) is 52%, while 13% in group (II), which is improved by 6% and 3%, respectively, compared with the second better approach in the average performance rank list; (2) AEI achieves the best average performance among all the methods in group (III). There is average 8% improvement in recognition rate by AEI; (3) The recognition performances of MSI and MEI are poorer than others. It is the reason that (1) GEI contains both static and dynamic information and achieves the best average recognition performance in group (I) and (II); (2) AEI has more dynamic information and attains the best average recognition performance in group (III). Moreover, there is no temporal information in MSI and MEI, which highlights the static information and dynamic information, respectively. The recognition performances of these two methods are worse than others.

The identification rates of the gait information introduction approach are summarized in Table 6. It can be seen from Table 6 that 1) MIEI has the best performance in group (I) and slightly improves the average recognition rate by 2% compared with EGEI, which is the second better approach in the average performance rank list. 2) We also notice that MIEI and EGEI perform better than other approaches in group (II). The average identification rate of MIEI in Rank1 is 13%. The Rank1 average identification rate of EGEI is 12%, which is 1% less than the performance of MIEI. While the MIEI losses 1% on rank5 performance compared with EGEI. 3) FDEI obtains the best average recognition performances in group (III). FDEI wins 3% and 4% on rank1 and rank5 performance, respectively, compared with CGI, which is the second better approach in the average performance rank list. 4) The recognition performances of GEnI and GFI are worse than the others. It is the reason that 1) MIEI, which is the weighted GEI, embodies more static information and achieves the highest average recognition rate in group (I). 2) The static and dynamic information have the equally important position in group (II). MIEI and EGEI have both static and dynamic information, and obtain better average identification performance. 3) FDEI has more dynamic information and attains the best average recognition performance in group (III). 4) GEnI and GFI are more sensitive to noise. Moreover, there is much noise for the silhouette images in the USF dataset, thus the recognition rate of GEnI and GFI is low.

The gait information introduction approach introduces some parameters, which are determined as follows. The whole gait sequence for MIEI should be analyzed. The pre-set average image *E*(*x*,*y*,*i*–1) and the weight *α* both affect the recognition performance. The average of the former 6 frames is chosen as the pre-set average image, and the value of *α* is 0.04 in the paper. The threshold parameter *T* of FDEI varies with different periods or subjects, we experimentally choose the value *T* as $0.6*\left(E_{\text{DEI}}^{c}\right)$ for each cluster in the gait recognition. However, FDEI is based on the addition of the segmented GEI and the difference image in the segment, the single difference image added slightly improves the recognition performance. Thus, we add the difference images and GEI of a whole gait cycle. The gamma correction tuning parameter *γ* plays an important role in EGEI. When *γ* is too big, the weight of DWM is much smaller and the useful information will be lost. When *γ* is too small, the weight of DWM is much bigger and there will be too much useless interfering information for recognition. Then, we choose the value of *γ* is equal to 0.9 in the paper.

The experimental results for the gait information fusion approach are illustrated in Table 7. The computational time of MMS is 78 s/f (second/frame), which is too long to meet the requirement of real time. There is no consideration of MMS in Table 7. The results shown in Table 7 indicate that 1) CGHI has the best performance in group (I), (II) and (III). 2) The recognition performance of X-T PEI is worse than the other. That is because that 1) CGHI has more information including the static, dynamic and temporal information. 2) X-T PEIs of different sequences need to be normalized. That is to say, the periods of different sequences should be compressed into the same. However, the static and dynamic information will be lost during normalization, thus the identification rate is lower than others.

The summary of several Class Energy Image approaches, which performs better, is recorded in Table 8. It can be seen from Table 8 that: 1) CGHI which belongs to the gait information fusion approach has higher identification rate than other representations in group (I) and (II). The rank1 and rank5 average identification rate of CGHI is improved by 3% and 5% respectively in group (II) compared with GEI. However, CGHI improves the accuracy slightly by 1% compared with MIEI in group (I). 2) AEI which belongs to the gait information accumulation approach achieves the best recognition performance in group (III). AEI wins 2% and 3% on rank1 and rank5 performance respectively compared with FDEI, which is the second better approach in the average performance rank list. It can be seen from the above analysis that CGHI and AEI are more robust for the noises of the silhouettes. Moreover, CGHI and AEI have better robustness to the external environment.

### 3.3. The Recognition Performance Analysis on CASIA B Dataset

The quality of the silhouettes in the CASIA B dataset is higher compared with that in the USF dataset. We perform experiments on CASIA B dataset to compare and analyze the performance of the Class Energy Image approaches under different walking states. As there are 10 gait sequences for each individual, we can adopt any one of them as the training data and generate one Class Energy Image for each individual, and use the remaining 9 gait sequences as testing data. Then, we employ 1-NN classifier (Rank1) to identify each testing gait sequence. Obviously, there are 10 × 9 = 90 different pairs of training data and testing data. To identify the influence of different environments, we categorize them into 9 groups according to the sampling environments.

The experimental results of the gait information accumulation approach, gait information introduction approach and gait information fusion approach are summarized in Table 9. The first column and row is different training and testing environments, respectively. The recognition rate in each cell is the average of all the experiments belonging to this group. For example, there are 12 experiments belonging to the case where the training environment is normal condition and the testing environment is walking on a coat.

It can be seen from App1 in Table 9 that 1) when we focus on the three groups on the diagonal, we can find that the recognition rate of GHI is higher than others in all the three groups. It means that when the training and testing environments are the same, the performance of GHI is better than others, and MSI is worst. 2) When we focus on all the six remaining groups, we can find that GHI wins in the three groups and fSHI wins in two groups. Furthermore, GHI improves the accuracy by almost 5% than fSHI on average. That means that GHI performs better and MHI has worse performance when the training and testing environments are different. Therefore, the results suggest that GHI is more robust for the external environment than the other methods belonging to the gait information accumulation approach.

The performances of the gait information introduction approach are provided in App2 (Table 9). Table App2 (Table 9) shows that 1) FDEI wins in all the three diagonal groups. It indicates that when the training and testing environments are the same, FDEI has higher recognition rate than others, and the performance of MIEI is the worst. 2) FDEI and CGI both win in three of all the six groups left. However, there is average 2.33% improvement by CGI compared with FDEI. It means that when the training and testing environments are different, CGI performs better than the others, and MIEI has worse performance. Therefore, it can be conveyed that CGI is more robust for the external environment.

Table App3 (Table 9) indicates that performances of CGHI outperform others in all the nine experimental groups. At the same time, X-T PEI has the worst performance. That is to say, in the gait information fusion approach, CGHI has more robustness for the external environment compared with the other methods.

The previous experiments in Section 3.3, under different training and testing environment, reveal that CGHI has a best performance among GHI, CGI and CGHI. In addition, in the same environment, all the Class Energy Image approaches attain comparable recognition accuracy. It is also worth comparing these approaches.

As the silhouette qualities in the USF are of higher noise than those in the CASIA B, we further explore the recognition rate of the Class Energy Images with respect to the different silhouette qualities. From the experimental results shown in Tables 6 and 9, we can see that the average identification rates of GFI, GEnI and CGI in the CASIA B are improved obviously, compared with those in the USF. Therefore, the above phenomenon illustrates that the silhouette qualities have significant influence on GFI, GEnI and CGI. That is to say, GFI, GEnI and CGI have no good robustness to the silhouette qualities.

## 4. Conclusions and Comments for Further Research

This paper has presented a comprehensive review of the video sensor-based gait representation methods, especially spatio-temporal motion summary approaches, namely Class Energy Image approaches. We have reviewed and analyzed various video-based Class Energy Image approaches, which have the following properties: (1) They contain rich motion information such as motion frequency, temporal and spatial changes of the human body; (2) They compress the information of a sequence to a template, which reduces the size of the gait database; (3) They are suitable for real time systems because Class Energy Image has a high computational efficiency; (4) They are insensitive to the quality of silhouettes and robust to silhouette errors or image noise. Based on the different ways of feature extraction and Class Energy Image generation, we divide the Class Energy Image approaches into three categories: the gait information accumulation approach, the gait information introduction approach and the gait information fusion approach. In summary, the gait information accumulation approach performs better and provides richer information than the original binary gait image. The disadvantage of this kind of method is that some useful information may be lost, and the problem of the inadequate training samples is raised; the gait information introduction approach can highlight dynamic information, meanwhile preserving the static information; the gait information fusion approach employs feature layer fusion and decision layer fusion method to achieve the fusion of static, dynamic and temporal information. Since the gait information fusion approach mainly takes different feature images into consideration, there is little correlation between different feature images. It is a promising direction to study the extraction of different features, and to fuse them using different fusion strategies. The experimental results demonstrated that some Class Energy Image approaches could attain higher recognition accuracy with good robustness and efficiency. Especially, the performance of CGHI is better than other templates. In addition, it is noteworthy that more studies should be implemented on the Class Energy Images with good performances, such as AEI, FDEI and CGI, *etc.*

We note that while significant successes have been achieved in his domain of research, some more work needs to be done as indicated next.

(1). As demonstrated in [1], extracting Gabor features from the gait energy images can further improve the performance of gait recognition. It is interesting to study whether the other invariant descriptors, such as Local Binary Pattern (LBP) [95] and Histogram of Oriented Gradients (HOG) [96] which have been demonstrated to benefit visual information processing and recognition in general, can further enhance the performance for the Class Energy Image-based gait recognition.
(2). The current gait information accumulation approach and gait information introduction approach highlight some gait information, but miss lots of information with discriminative faculties. Experimental results reveal that CGHI, which belongs to the gait information fusion approach, outperforms other Class Energy Images. The reason for this phenomenon is that CGHI contains richer information (dynamic, static and temporal information). This provides a new insight to gait recognition. We could derive more gait features by implementing feature-level fusion for different Class Energy Images. The gait features to be fused could be selected from the gait information accumulation approach and gait information introduction approach.
(3). Gait is sensitive to various covariate conditions such as view angle, speed, clothing, carrying condition (backpack, briefcase, handbag, *etc.*), shoe-wear type, surface, accessories, injury, mood and to name a few. The further researches of Class Energy Image approach should take all these conditions into consideration and new approaches with robustness and efficiency should be presented.
(4). The Class Energy Image approaches can also be applied to other biometric identification areas such as multi-pose face recognition. Several multi-pose face images from the same individual could be integrated to an image by using Class Energy Image approaches. In order to achieve the real-time performance, the representations of biometric features should be as simple as possible, and the computational complexity will be as low as possible.

## Figures and Tables

**Table 1.** The information of the gait information accumulation approach.

**Name**	**Expression**	*O*	**Dynamic**	**Static**	**Time**	**Image**	
MEI	$\begin{array}{l} E_{\text{MEI}}\left(x,y,n\right)=\cup_{i=0}^{\tau -1}D\left(x,y,n-i\right) \end{array}$	*O*(*η*·*m*·*n*)	√	×	×	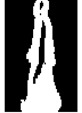	
MHI	$\begin{array}{c} E_{\text{MEI}}\left(x,y,n\right)= \end{array}$	*O*(*η*·*m*·*n*)	√	×	√	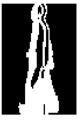	
MSI	$\begin{array}{c} E_{\text{MSI}}\left(x,y,n\right)= \end{array}$	*O*(*η*·*m*·*n*)	×	√	√	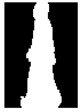	
GEI	$\begin{array}{l} E_{\text{GEI}}=\frac{1}{N}\overset{N}{\underset{n=1}{\text{∑}}}B\left(x,y,n\right) \end{array}$	*O*(*η*·*m*·*n*)	√	√	×	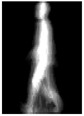	
GHI	$\begin{array}{c} E_{\text{GHI}}\left(x,y\right)= \end{array}$	*O*(*η*·*m*·*n*)	√	√	√	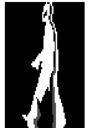	
fSHI	$\begin{array}{l} E_{\text{fSHI}}\left(x,y\right)=\overset{N_{\lambda }-1}{\underset{n=1}{\text{∑}}}E_{\text{fSHI}}\left(x,y,n\right)\\ E_{\text{fSHI}}\left(x,y,n\right)=\{\begin{array}{ll} n\tau_{1} & \text{if}\;D\left(x,y,n\right)=1\\ 0 & \text{otherwise} \end{array} \end{array}$	*O*(*η*·*m*·*n*)	√	×	√	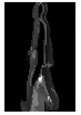	
bSHI	$\begin{array}{l} E_{\text{bSHI}}\left(x,y\right)=\overset{N_{\lambda }-1}{\underset{n=1}{\text{∑}}}E_{\text{fSHI}}\left(x,y,n\right)\\ E_{\text{bSHI}}\left(x,y,n\right)=\{\begin{array}{ll} n\tau_{1} & \text{if}\;D\left(x,y,n\right)=-1\\ 0 & \text{otherwise} \end{array} \end{array}$	*O*(*η*·*m*·*n*)	√	×	√	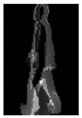	
AEI	$E_{\text{AEI}}=\frac{1}{N}\overset{N}{\underset{n=1}{\text{∑}}}D_{n}\left(x,y,n\right)$	*O*(*η*·*m*·*n*)	√	×	×	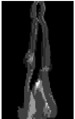	
GMI	$E_{\text{GMI}}\left(x,y,k\right)=\frac{1}{C}\overset{C}{\underset{i=1}{\text{∑}}}B_{i}\left(x,y,k\right)$	*O*(*C*·*S*·*m*·*n*)	√	√	×	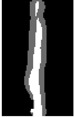	
MGEI	$\begin{array}{l} E_{M\text{GEI}}\left(x,y,k\right)=\frac{1}{2\times \sum_{i=1}^{\overset{S}{\;}/\underset{2}{\;}}r^{i}}\sum_{i=1}^{\overset{S}{\;}/\underset{2}{\;}}\sum_{i=-1,1}r_{1}^{i}\\ \times \frac{1}{C}\sum_{C=1}^{C}B\left(x,y,\left(k+S+i\times i\right)\%S\right) \end{array}$	*O*(*C*·*S*^2^·*m*·*n*)	√	√	×	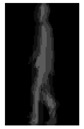	
GDI	$\begin{array}{l} E_{\text{GDI}}\left(x,y,k\right)=\{\begin{array}{cc} \max(E_{\text{GDI}}\left(x,y,k-1\right)-255/N,0) & \text{if}\;E_{\text{DGMI}}\left(x,y,k\right)=0\\ \max\left(E_{\text{GDI}}\left(x,y,k-1\right),E_{\text{DGMI}}\left(x,y,k\right)\right) & \text{else} \end{array}\\ E_{\text{DGMI}}\left(x,y,k\right)=\frac{1}{C}\overset{C}{\underset{i=1}{\text{∑}}}\left|B_{i}\left(x,y,k\right)-E_{M\text{GEI}}\left(x,y,k\right)\right| \end{array}$	*O*(*C*^2^·*S*^2^·*m*·*n*)	√	√	×	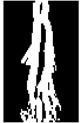	

Note: We make √ and × represent whether the Class Energy Image with or without the type of the motion information, respectively. *O* denotes computational complexity. Suppose the size of the silhouette is *m*×*n*, *η* is the numbers of silhouettes in a gait cycle.

**Table 2.** The information of the gait information introduction approach.

**Name**	**Expression**	*O*	**Dynamic**	**Static**	**Time**	**Image**
MIEI	*E_i_* = *α* × *B*_*i*−1_ + (1 − *α*) × *E*_*i*−1_, *α* ∈ (0,1)	*O*(*η*·*m*·*n*)	√	√	√	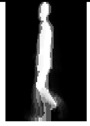
FDEI	$\begin{array}{l} E_{\text{FDEI}}\left(x,y\right)=F\left(x,y,n\right)+E_{\text{DEI}}^{c}\left(x,y\right)\\ {E^{c}}_{\text{DEI}}\left(x,y\right)=\{\begin{array}{ll} \frac{1}{N_{c}}\underset{n\in N_{C}}{\text{∑}}B\left(x,y,n\right) & \text{if}\frac{1}{N_{c}}\underset{n\in N_{C}}{\text{∑}}B\left(x,y,n\right)\geq T\\ 0 & \text{otherwise} \end{array}\\ F\left(x,y,n\right)=\{\begin{array}{ll} 0 & \text{if}\;B\left(x,y,n\right)\geq B\left(x,y,n-1\right)\\ B\left(x,y,n-1\right)-B\left(x,y,n\right) & \text{otherwise} \end{array} \end{array}$	*O*(*η*·*m*·*n*)	√	√	×	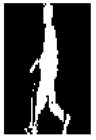
EGEI	$\begin{array}{l} E_{\text{EGEI}}\left(x,y\right)=G\left(x,y\right)\times {\left(T_{\text{DWN}}\left(x,y\right)\right)}^{\gamma }\\ \sigma_{\text{GEI}}\left(x,y\right)=\sqrt{\frac{1}{A}\overset{A}{\underset{i=1}{\text{∑}}}{\left[\frac{1}{N}\overset{N}{\underset{m=1}{\text{∑}}}G_{m}^{i}\left(x,y\right)-\frac{1}{A}\overset{A}{\underset{i=1}{\text{∑}}}\frac{1}{N}\overset{N}{\underset{m=1}{\text{∑}}}G_{m}^{i}\left(x,y\right)\right]}^{2}} \end{array}$	*O*(*A*^2^·*η*·*m*·*n*)	√	√	×	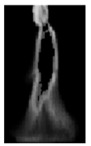
CGI	$E_{\text{CGI}}\left(x,y\right)=\frac{1}{p}\overset{p}{\underset{i=1}{\text{∑}}}\sum_{t=1}^{n_{i}}C_{t}\left(x,y\right)$	*O*(*p*·*η_i_*·(*m*·*n*)^2^)	√	√	√	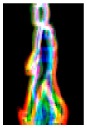
GFI	$\begin{array}{l} E_{\text{GFI}}\left(x,y\right)=\frac{\sum_{n=1}^{N-1}F_{n}\left(x,y\right)}{N}\\ F_{i}\left(x,y\right)=\{\begin{array}{ll} 0 & \text{if}\;\text{Mag}F_{i}\left(x,y\right)\geq 1\\ 1 & \text{otherwise} \end{array}\\ \text{Mag}F_{i}\left(x,y\right)=\sqrt{{\left(\mu F_{i}\left(x,y\right)\right)}^{2}+{\left(\nu F_{i}\left(x,y\right)\right)}^{2}} \end{array}$	*O*(*η*·(*m*·*n*)^2^)	√	×	√	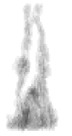
GEnI	$E_{\text{GEnI}}\left(x,y\right)=-E_{\text{GEI}}\left(x,y\right)\log_{2}E_{\text{GEI}}\left(x,y\right)-\left(1-E_{\text{GEI}}\left(x,y\right)\right)\log_{2}\left(1-E_{\text{GEI}}\left(x,y\right)\right)$	*O*(*η*·*m*·*n*)	√	√	×	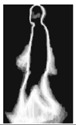

Note: We make √ and × represent whether the Class Energy Image with or without the type of the motion information, respectively. *O* denotes computational complexity. Suppose the size of the silhouette is *m*×*n*, *η* is the numbers of silhouettes in a gait cycle.

**Table 3.** The information of the Gait information fusion approach.

**Name**	**Expression**	*O*	**Dynamic**	**Static**	**Time**	**Image**
X-T PEI	$E_{X-T\text{PEI}}\left(x,y\right)=\frac{1}{H}\overset{H}{\underset{y=1}{\text{∑}}}B\left(x,y,n\right)$	*O*(*η*·*m*·*n*)	√	√	×	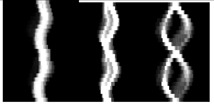
CGHI	$\begin{array}{l} I_{R}\left(x,y,1)=E_{{\text{fSHI}}_{1}}(x,y\right)\\ I_{G}\left(x,y,2)=E_{{\text{fSHI}}_{2}}(x,y\right)\\ I_{B}\left(x,y,3)=E_{\text{GEI}}(x,y\right) \end{array}$	*O*(*η*·*m*·*n*)	√	√	√	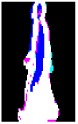
MSCT and SST	$\begin{array}{l} E_{\text{MSCT}}\left(x,y,n\right)=\{\begin{array}{cc} 255 & \text{if}\;A_{i}\left(x,y,n\right)=1\\ \max\left(0,A_{i}\left(x,y,n\right)-\frac{255}{P}\right) & \text{otherwise} \end{array}\\ E_{\text{SST}}\left(x,y,n\right)=\{\begin{array}{ll} 1 & ifE_{\text{SST}}\left(x,y,n\right)=E_{\text{SST}}\left(x,y,n-1\right)\\ 0 & \text{otherwise} \end{array}\\ A_{i}\left(x,y,n\right)=B_{i}\left(x,y,n\right)-\underset{s\in S}{\cap }{\left(B_{i}\left(x,y,n\right)\right)}_{-s} \end{array}$	*O*(*η*·*m*·*n*)	√	√	×	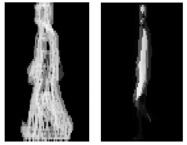
AME and MMS	$\begin{array}{l} E_{\text{AME}}=\frac{1}{N}\overset{n}{\underset{n=1}{\text{∑}}}B_{n}\left(x,y,n\right)\\ E_{\text{MMS}}=\overset{n}{\underset{j=1}{\text{∑}}}\left(S_{j}{S_{j}}^{\text{T}}\right)/\left({S_{j}}^{\text{T}}S_{j}\right) \end{array}$	*O*(*η*·(*m*·*n*)^2^)	√	√	×	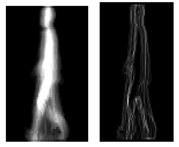

Note: We make √ and × represent whether the Class Energy Image with or without the type of the motion information, respectively. *O* denotes computational complexity. Suppose the size of the silhouette is m×n, *η* is the numbers of silhouettes in a gait cycle.

**Table 4.** The USF Database.

**Dateset**	**Number of Samples**	**Variations**	**Dateset**	**Number of Samples**	**Variations**
Gallery	122	G,A,R,NB	— — —	— — —	— — —
Probe A	122	G,A,L,NB	Probe G	60	C,B,L,NB
Probe B	54	G,B,R,NB	Probe H	120	G,A,R,BF
Probe C	54	G,B,L,NB	Probe I	60	G,B,R,BF
Probe D	121	C,A,R,NB	Probe J	120	G,A,L,BF
Probe E	60	C,B,R,NB	Probe K	33	G,A/B,R,NB,T
Probe F	121	C,A,L,NB	Probe L	33	C,A/B,R,NB,T

**Table 5.** The recognition performances of the gait information accumulation approach (%).

**Group**	**Probe**	**Variation**	**MEI**		**GHI**		**MHI**		**fSHI**		**bSHI**		**MSI**		**GEI**		**AEI**	

			**Rank1**	**Rank5**	**Rank1**	**Rank5**	**Rank1**	**Rank5**	**Rank1**	**Rank5**	**Rank1**	**Rank5**	**Rank1**	**Rank5**	**Rank1**	**Rank5**	**Rank1**	**Rank5**
I	A	view	14	48	6	18	30	61	31	56	33	63	25	47	56	81	53	75
	B	show	6	9	9	50	33	50	48	65	48	66	30	63	67	80	54	78
	C	view, shoe	2	13	4	9	15	30	13	37	20	43	11	28	32	57	32	56
	Avg.	--	7	23	6	26	26	47	20	53	34	57	22	46	***52***	73 *	46	70

II	D	surface	7	28	1	8	4	19	7	21	9	23	9	18	15	40	10	26
	E	surface, shoe	2	7	0	5	5	8	12	18	10	22	10	5	15	38	15	23
	F	surface, view	3	20	1	7	3	12	4	17	5	16	7	18	8	27	8	22
	G	surface, shoe, view	0	13	0	5	5	8	7	18	7	18	2	13	13	27	7	20
	Avg.	--	4	17	1	6	4	12	8	19	8	20	7	14	***13***	33 *	10	23

III	H	briefcase	7	45	5	21	3	12	31	54	36	58	26	55	33	64	48	66
	I	briefcase, shoe	3	8	3	17	18	35	23	47	33	47	25	50	33	67	45	73
	J	briefcase, view	9	43	3	13	13	32	16	39	20	38	18	36	24	53	30	52
	K	time, shoe, clothing	0	3	0	3	0	3	0	9	0	12	3	18	3	6	9	27
	L	surface, time, shoe, clothing	0	6	0	6	3	12	6	9	6	21	15	24	3	3	3	15
	Avg.	--	4	33	2	12	9	19	15	32	19	35	17	37	19	39	***27***	47 *

Note: ***A*** and A* (***A*** is number) denote the best Rank 1 and Rank 5 performances, respectively.

**Table 6.** The recognition performances of the gait information introduction approach (%).

**Group**	**Probe**	**variation**	**MEI**		**FDEI**		**EGEI**		**CGI**		**GFI**		**GEnI**	

			**Rank 1**	**Rank 5**	**Rank 1**	**Rank 5**	**Rank 1**	**Rank 5**	**Rank 1**	**Rank 5**	**Rank 1**	**Rank 5**	**Rank 1**	**Rank 5**
I	A	view	56	83	53	75	56	82	43	73	49	75	35	59
	B	show	70	82	44	70	67	81	51	83	41	57	39	76
	C	view, shoe	33	61	30	50	30	60	30	54	24	52	17	46
	Avg.	--	***53***	75 *	42	65	51	74	41	70	38	61	30	60

II	D	surface	17	41	12	23	13	41	18	36	10	22	12	27
	E	surface, shoe	17	35	7	25	13	40	12	32	13	25	12	32
	F	surface, view	7	28	4	18	8	28	7	26	9	21	7	24
	G	surface, shoe, view	12	30	5	17	12	30	8	25	12	17	3	17
	Avg.	--	***13***	34 *	7	21	***12***	35 *	11	30	11	21	9	25

III	H	briefcase	32	61	46	63	34	70	37	66	39	63	33	62
	I	briefcase, shoe	33	63	43	70	33	68	45	62	40	63	28	57
	J	briefcase, view	28	53	25	52	27	55	23	44	23	52	25	41
	K	time, shoe, clothing	0	6	6	18	3	6	0	6	0	15	0	21
	L	surface, time, shoe, clothing	6	6	6	15	3	12	6	24	3	15	15	24
	Avg.	--	20	38	***25***	44 *	20	42	22	40	21	42	20	41

**Note: *A*** and A* (***A*** is number) denote the best Rank1 and Rank5 performances, respectively.

**Table 7.** The recognition performances of the gait information fusion approach (%).

**Group**	**Probe**	**Variation**	**X-T PEI**		**MSCT&SST**		**CGHI**		**AME&MMS**	

			**Rank 1**	**Rank 5**	**Rank 1**	**Rank 5**	**Rank 1**	**Rank 5**	**Rank 1**	**Rank 5**
I	A	view	36	29	53	82	60	84	/	/
	B	show	51	60	69	80	68	82	/	/
	C	view, shoe	28	45	30	55	35	61	/	/
	Avg.	--	38	45	51	72	***54***	76 *	/	/

II	D	surface	4	12	8	52	18	45	/	/
	E	surface, shoe	3	15	3	20	19	44	/	/
	F	surface, view	2	16	8	21	11	32	/	/
	G	surface, shoe, view	10	15	9	15	17	33	/	/
	Avg.	--	5	15	7	27	***16***	39 *	/	/

III	H	briefcase	15	53	20	60	37	67	/	/
	I	briefcase, shoe	18	59	23	64	38	71	/	/
	J	briefcase, view	20	40	25	45	28	58	/	/
	K	time, shoe, clothing	21	6	24	40	6	9	/	/
	L	surface, time, shoe, clothing	0	6	6	9	9	12	/	/
	Avg.	--	15	33	20	44	***22***	43 *	/	/

**Note: *A*** and A* (***A*** is number) denote the best Rank 1 and Rank 5 performances, respectively.

**Table 8.** Several best average recognition performance approaches (%).

**Group**	**GEI**		**AEI**		**MIEI**		**FDEI**		**CGHI**	

	**Rank 1**	**Rank 5**	**Rank 1**	**Rank 5**	**Rank 1**	**Rank 5**	**Rank 1**	**Rank 5**	**Rank 1**	**Rank 5**
I	52	73	/	/	53	75	/	/	54	76
II	13	34	/	/	13	33	/	/	16	39
III	/	/	27	47	/	/	25	44	22	43

**Table 9.** The Rank 1 performance of Class Energy Image (%).

**Test**	**Train**											

	**App1**				**App2**				**App3**			

		**Normal**	**Bag**	**Coat**		**Normal**	**Bag**	**Coat**		**Normal**	**Bag**	**Coat**
Normal	MEI	86	62	37	MIEI	78	26	33	X-T PEI	69	31	34
	GHI	***96***	65 *	33	FDEI	***94***	68 *	42	MSCT &SST	93	49	57
	MHI	72	31	32	EGEI	87	58	38	CGHI	***95***	***58***	60 *
	fSHI	94	50	39	CGI	87	63	53 *				
	bSHI	91	54	46 *	GFI	86	54	45				
	MSI	78	40	39	GEnI	92	62	44				
	GEI	90	44	26								
	AEI	93	54	34								

Bag	MEI	25	85	59	MIEI	17	75	17	X-T PEI	24	68	14
	GHI	42 *	***98***	66	FDEI	39	***98***	44 *	MSCT &SST	51	95	44
	MHI	12	68	9	EGEI	39	90	19	CGHI	69 *	***98***	64 *
	fSHI	42 *	91	69 *	CGI	45 *	95	40				
	bSHI	40	98	44	GFI	41	93	26				
	MSI	24	65	30	GEnI	39	93	27				
	GEI	31	90	17								
	AEI	38	93	29								

Coat	MEI	11	50	86	MIEI	19	20	85	X-T PEI	15	11	88
	GHI	25	79 *	***97***	FDEI	22	56 *	***98***	MSCT &SST	34	35	96
	MHI	27	16	88	EGEI	22	27	95	CGHI	48 *	55 *	98
	fSHI	36	45	93	CGI	43 *	41	95				
	bSHI	40 *	52	93	GFI	33	32	96				
	MSI	32	38	73	GEnI	32	32	91				
	GEI	27	20	96								
	AEI	38	31	93								

**Note: *A*** and A* (***A*** represents number) represents the best performance data. App1 represents the gait information accumulation approach. App2 represents the gait information introduction approach. App3 represents the gait information fusion approach.
